# Peripheral primitive neuroectodermal tumor associated with paraneoplastic Cushing's syndrome: The rare case

**DOI:** 10.1016/j.amsu.2018.11.018

**Published:** 2018-11-29

**Authors:** Mahtab Rahbar, Maryam Rahbar, Gholamreza bahoush

**Affiliations:** a Iran University of Medical Science, Tehran, Iran; b Tehran Medical Science of University, Tehran, Iran

**Keywords:** Peripheral primitive neuroectodermal tumor, Cushing's disease, Paraneoplastic syndrome

## Abstract

**Introduction:**

Primitive neuroectodermal tumors (PNET) form a group of tumors defined by their appearance that are thought to develop from primitive (undifferentiated) nerve cells in the brain. They are rare tumors and their incidence is not well defined.

**Case presentation:**

An 18-month-old male presenting with typical Cushingoid appearance (moon face, central obesity, hirsutism and growth arrest) was admitted for evaluation of endocrine problems. Subsequent laboratory studies revealed markedly elevated adrenocorticotropic (ACTH) and cortisol levels, as well as a hypokalemic metabolic alkalosis, these data are consistent with the diagnosis of Cushing's disease. He was treated with metyrapone to control hypercortisolemia. One month and a half later, a mass was detected in the abdomen by ultrasonography. An abdominal Computed tomography confirmed a large heterogeneous retroperitoneal mass with a significant amount of extension into surrounding structures which was removed by laparoscopic abdominal surgery. The patient's symptoms completely resolved and the ACTH and cortisol levels were also greatly reduced. Histologically, the tumor tissue consistent with the diagnosis of the retroperitoneal primitive neuroectodermal tumor which was confirmed immunohistochemically. This case demonstrates the successful diagnosis and treatment of a rare neoplasm.

**Conclusion:**

This is the first rare case with ectopic ACTH syndrome caused by the peripheral primitive neuroectodermal tumor.

## Highlights

• Paraneoplastic Cushing's syndrome has been attributed to ectopic ACTH secretion.
• Primitive neuroectodermal tumor secret ectopic ACTH and make Cushing's syndrome.
• Our case is an 18-month-old male presenting Cushing's syndrome because of retroperitoneal primitive neuroectodermal tumor.
• After remove of tumor,the patient's symptoms completely resolved.

## 1 Introduction

Peripheral primitive neuroectodermal tumor (PNET) is an uncommon tumor and the overall incidence is 1% of all sarcomas [1]. Primitive neuroectodermal tumors (PNETs) are rare small round cell neoplasms, first described in 1918 by Stout [2] as a malignant tumor arising from major nerve [3]. This tumor can occur at any age, although the peak age incidence is during adolescence and young adulthood and the tumors are rarely observed in children <3 years old. The incidence of peripheral PNET in the abdomen and pelvis, including the retroperitoneum, is about 14% of all peripheral PNETs [4]. Cushing's syndrome is a disorder of hypercortisolism which has a characteristic appearance (moon facies, buffalo hump, truncal obesity, and purple striae). Furthermore, it is associated with a variety of organ dysfunctions including cardiovascular, endocrine, neural, gastrointestinal, skin, and musculoskeletal. Ectopic secretion of ACTH accounts for ∼10% of Cushing's syndrome etiologies [[5], [6], [7], [8]].The following case is that of an 18 months-old Iranian boy presenting with Cushing's syndrome due to ACTH-secreting peripheral PNET. Our case is very rare in its presentation.

## 2 Case presentation

**Fig. 1:**
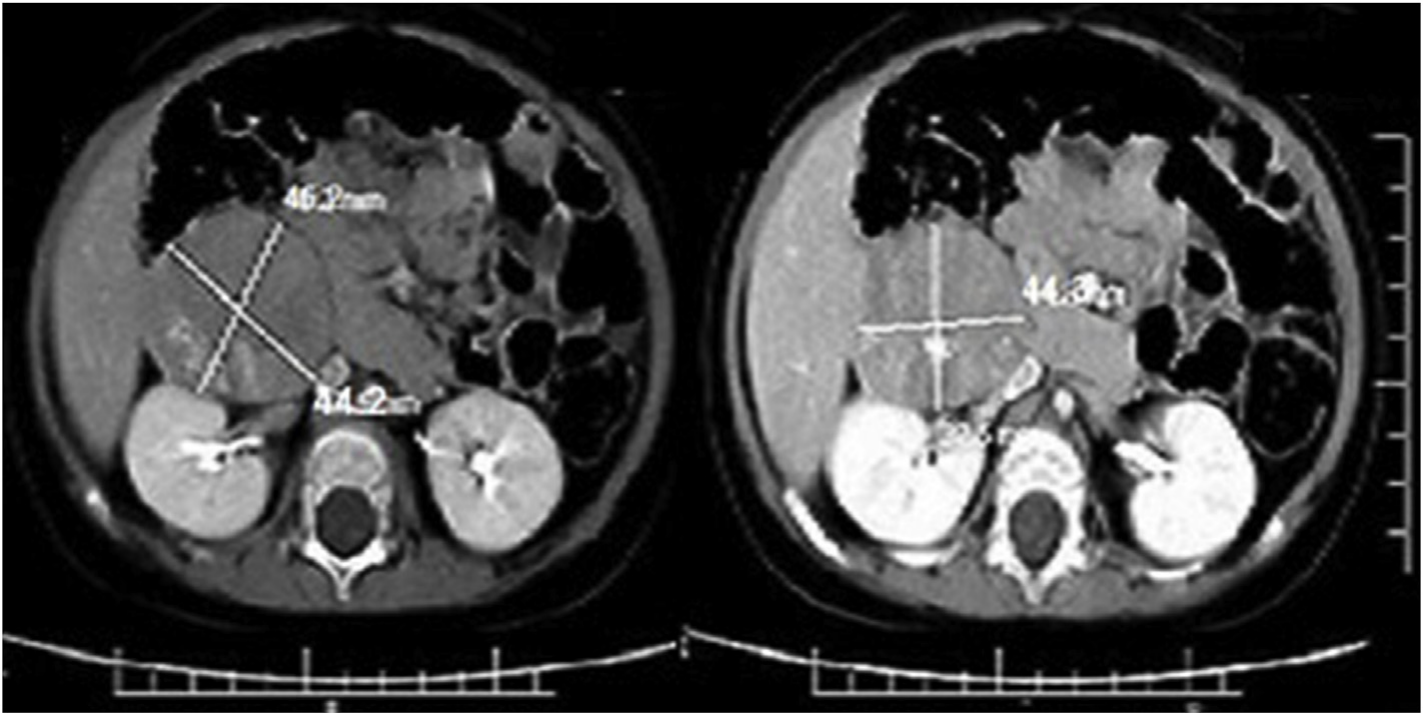
Contrast enhanced computed tomography abdomen showed large lobulated necrotic hypodense enhancing lesion involving part of retroperitoneal space.

**Fig. 2:**
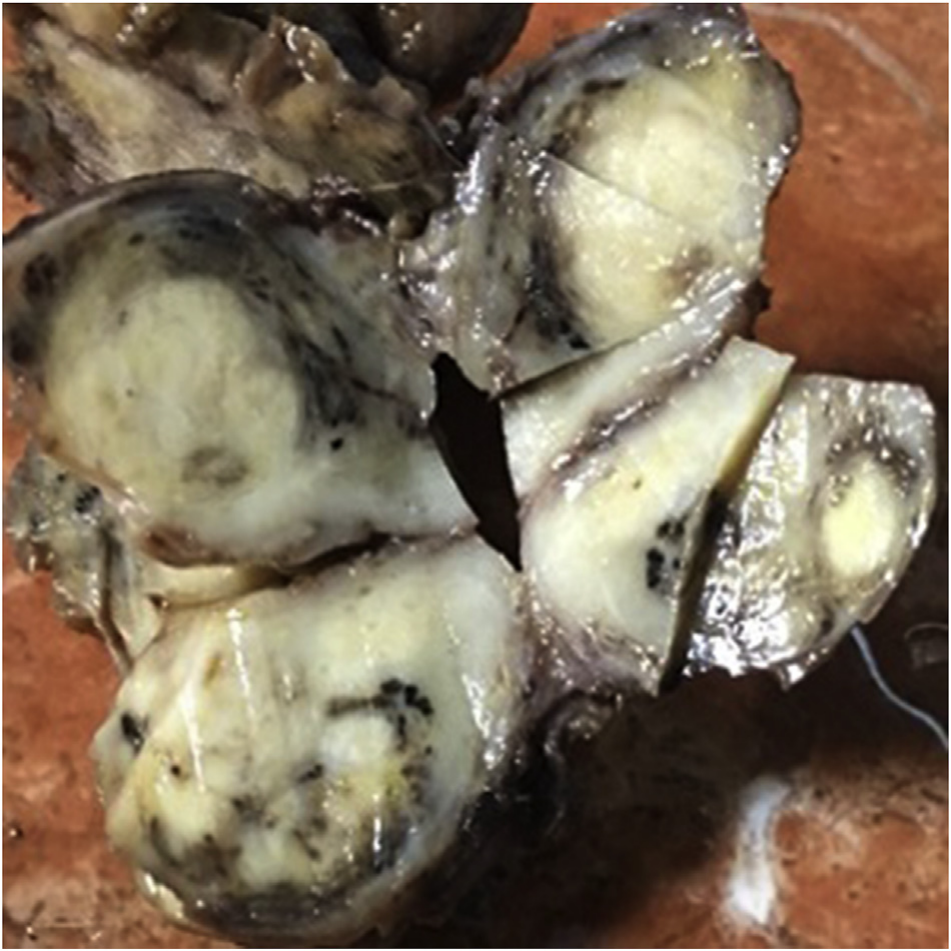
Gross image of a renal primitive neuroectodermal tumor. This specimen is notable for a variegated appearance. Select areas of the tumor feature a tan/brown or dark brown/red coloration, whereas other sections of the tumor feature a more yellow appearance, helping to illustrate the range of coloration observable on gross examination. This specimen is also notable for its lobulated appearance.

**Fig. 3:**
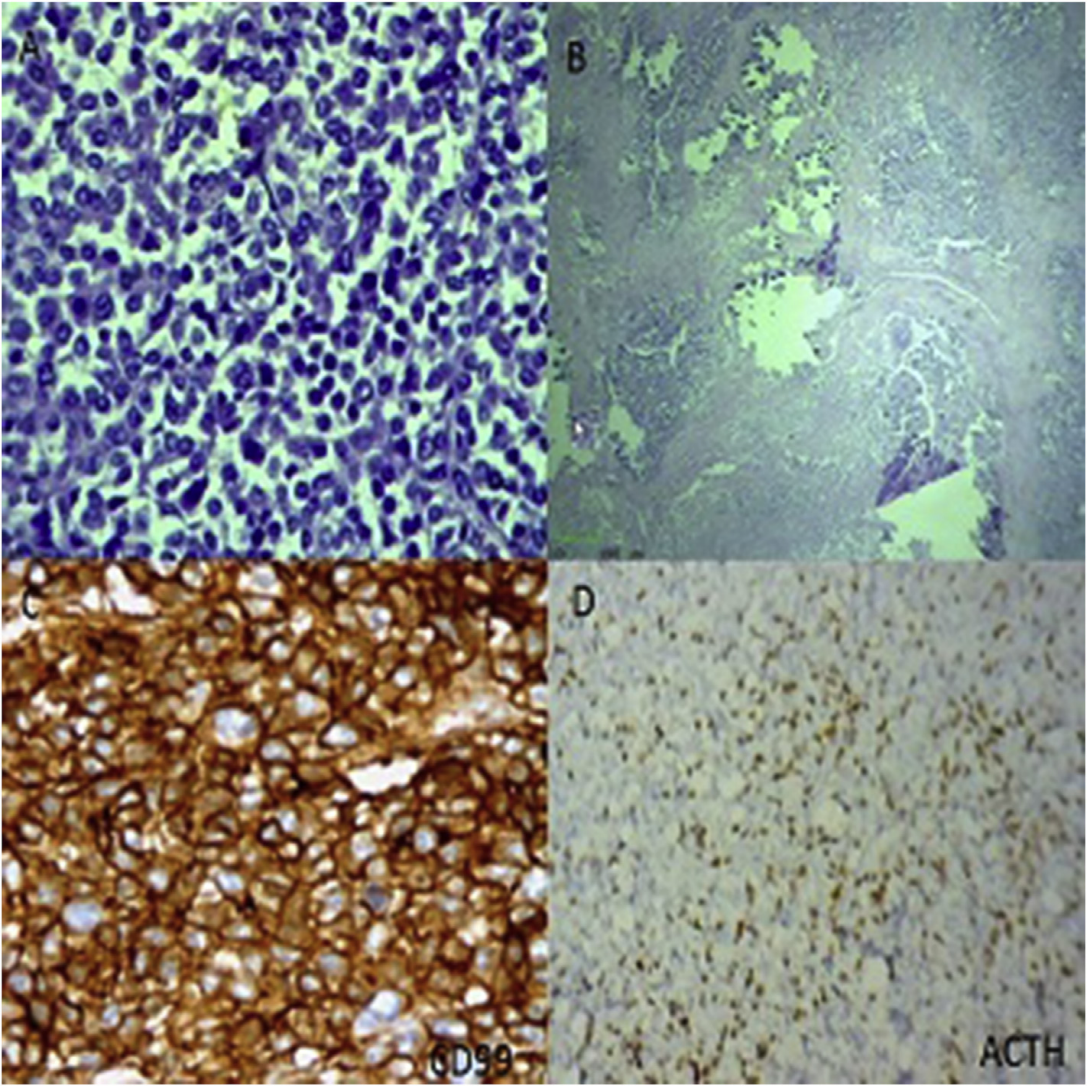
A, Hematoxylin and eosin features of the tumor (original magnification 40 ×): sheets of poorly differentiated, monotonous, ovoid cells, with a high nuclear to cytoplasmic ratio. Homer-Wright rosettes were common. B, Tumor cells with necrotic areas and foci of calcifications .C, Immunoperoxidase for CD99 (40×) showed predominantly membranous staining of tumor cells composed of small round cells with round nuclei and scant cytoplasm arranged in cohesive lobules. D, Immunoperoxidase for ACTH (10×) showed Diffuse cytoplasmic staining of tumor cells.

In september2017, an 18-month-old male admitted in endocrine department with symptoms of moon face, general weakness, central obesity, growth arrest and short stature of 5 months duration. On physical exam, the patient was noted to have 1 + pitting edema on his lower extremities bilaterally and hirsutism in back of trunk. At the time of presentation, he did not appear severely cushingoid appearance and his blood pressure was (115/61 mm Hg). Laboratory results revealed highly elevated ACTH and cortisol levels (ACTH = 731 pg/mL; AM cortisol = 142.8 μg/dL; 24-h urine cortisol 12743.5μg/24 hours total volume). In addition, the patient was also hypokalemic (3.0 mEq/L) and had a metabolic alkalosis (pH = 7.89, HCO3 = 41 mEq/L). Dexamethasone suppression test was considered: however, in the presence of very high ACTH and cortisol levels, hypokalemia, and metabolic alkalosis, as well as clinical findings, a primary pituitary tumor or an ectopic ACTH syndrome was suspected. Brain MRI was negative for primary pituitary tumor. Abdominal ultrasonography (USG) showed a solid, calcified heterogeneous mass measuring 57 × 46 × 36 mm in front of anteromedial of right kidney near to inferior pole. A Doppler ultrasound test showed the blood flow through inferior vena cava (IVC) restricted by pressure effect of tumor. Contrast-enhanced computed tomography abdomen showed large lobulated, necrotic and calcified hypodense enhancing mass measuring 47.6 × 44.3 mm in the largest diameters that extending from anterior of right kidney to retroperitoneum and involving retroperitoneal space. The lesion was abutting the IVC, displacing it laterally (Fig. 1) The USG/CT appearance of the mass, in combination with the clinical and laboratory findings, was suspicious for neuroblastoma or pPNET. Informed consent was obtained from parents prior to surgery. Total resection of mass was performed by laparoscopic abdominal surgery. After surgery, his metabolic abnormalities were controlled. Grossly, the tumor was brownish, soft and multilobulated. Gross examination of the specimen sent to us revealed a soft tissue mass measuring about 47 × 42 × 37 mm, cut surface of which revealed an encapsulated creamy to brownish mass with lobulated and variegated appearance with solid and necrotic areas with foci of calcification (Fig. 2). On microscopic examination, there were lobules of tumor cells separated by fibrous septa. The tumor cells comprised of small cells with round vesicular nuclei, inconspicuous nucleoli, scanty eosinophilic cytoplasm with frequent homer wright rosettes and pseudorosettes (Fig. 3). The immunohistochemical evaluation revealed a diffuse CD99 and vimentin positivity in the cytoplasm of the neoplastic cells. Pan keratin, cytokeratin AE1/AE3, myogenin and CD45 were negative. The S-100 protein was weakly positive in tumor cells. Ki67 immunostain shows about 65% immunoreactivity. Also, there were focal expression of synaptophysin and ACTH (Fig. 3). These findings represent an ACTH-secreting PNET. Following the operation, his ACTH level decreased to 22 pg/mL. Ki67 immunostain shows about 65% immunoreactivity. He was discharged on hydrocortisone 5 mg in the morning and 5mg in the evening for secondary glucocorticoid deficiency because of prolonged ectopic ACTH secretion. When the patient finished the hydrocortisone course, he maintained a normal ACTH, morning plasma cortisol, and urine cortisol levels. Furthermore, his Cushing's syndrome symptoms completely resolved.

## 3 Discussion

Approximately 5%–10% of cases of Cushing syndrome (hypercortisolism) are paraneoplastic.[9] Paraneoplastic Cushing's syndrome has been attributed to ectopic adrenocorticotropin (ACTH) secretion, which may result in several types of tumors, including carcinoid and pancreatic neuroendocrine tumors [10], small cell lung cancer and thyroid medullary carcinoma [11], Wilms tumor [12], prostatic cancer [13,14], neuroblastoma [11], and thymus tumor [15,16]. Patients often present with symptoms of paraneoplastic Cushing syndrome before a cancer diagnosis is made. Similarly, relapse of paraneoplastic Cushing syndrome may herald tumor recurrence [12].Paraneoplastic Cushing syndrome arises from tumor secretion of adrenocorticotropic hormone or corticotropin-releasing factor [9,13]. The diagnosis of ectopic ACTH syndrome secondary to esthesioneuroblastoma was established based on the presence of ACTH seen on immunohistochemical staining of the tumor, the disappearance of symptoms, as well as a decrease and normalization of plasma ACTH and cortisol levels after resection of the tumor. Peripheral primitive neuroectodermal tumor (pPNET) is a group of malignant small round cell tumor with neural crest origin that arises outside the central and sympathetic nervous system [17].They belong to the Ewing sarcoma family of tumors with similar histologic and immunohistochemical characteristics [18]. Peripheral PNETs are rare in the abdomen [17]. This tumor rarely presents as an organ-derived neoplasm [19]. Carefully the selected immunohistochemical panel is important for differentiating this tumor from other small round cell tumors in abdomens such as rhabdomyosarcoma, neuroblastoma, clear cell sarcoma of the kidney, non-Hodgkin lymphoma and Ewing's sarcoma. Immunohistochemically, PNET cells express vimentin, NSE, and CD99 [20]. The positive reactivity to CD99 is a clue for PNET diagnosis [19]. Review of radiologic findings showed that most cases of retroperitoneal PNETs in the literature, similar to our case, presented as a large mass with areas of necrosis and heterogeneous enhancement in contrast imaging. Some cases had areas of hemorrhage, but in our case, there was no hemorrhage. Our case had several foci of curvilinear calcification compared to other case reports, in which calcification was a rare finding (one of ten cases in a case series) [21]. Organ invasion and displacement, such as direct invasion or displacement of the pancreas, kidney, spleen, and stomach was present in some cases, including our case but our case didn't show [21].There was no clinical or imaging evidence of venous thrombosis in the inferior vena cava (IVC), while venous thrombosis has been detected in other patients [20,21]. Approximately 20% of the patients present with metastatic disease; out of which 44% present with lung metastases only, 51% have bone or bone marrow involvement (with or without lung metastases) and less than 5% present with metastases in other organs. There is no evidence of invasion or metastasis in our case. Also, splitting calcification is seen rarely [22]. However, our case was associated with Cushing's syndrome which was a paraneoplastic syndrome. Thus, this is the first rare case with ectopic ACTH syndrome caused by the peripheral primitive neuroectodermal tumor. Ectopic ACTH syndrome due to pPNET is extremely uncommon with no case being discussed in the literature.

## 4 Conclusion

pPNET associated with Cushing's syndrome is extremely rare. This is the first report that didn't report cases in the literature. This report intends to highlight that PNET should be suspected whenever a patient presents with paraneoplastic Cushing's syndrome and may often be confused with other tumors presented with the cushingoid syndrome. Early diagnosis is important to ensure timely surgical resection followed by appropriate chemotherapy and/or radiotherapy.

## Patient consent

The parents have given consent for possible publication of this case report and accompanying images.

## Provenance and peer review

Not commissioned, externally peer reviewed.

## Ethical approval

Ethics approval was not applicable.

## Sources of funding

No external funding was required for this study.

## Author contribution

Mahtab rahbar and maryam rahbar: Data collection, data analysis & interpretation and writing the paper. Gholamreza Bahoush: Study concept & design, data collection, data analysis or interpretation, writing the paper.

## Conflicts of interest

The authors declare that they have no competing interests.

## Trial registry number

Research Registry number:4291.

## Guarantor

Mahtab Rahbar.

## Appendix A Supplementary data

SCARE Guideline Checklist,ijscr SCARE Guideline Checklist,ijscr
